# The Accessibility, Usability, and Reliability of Chinese Web-Based Information on HIV/AIDS

**DOI:** 10.3390/ijerph13080834

**Published:** 2016-08-20

**Authors:** Lu Niu, Dan Luo, Ying Liu, Shuiyuan Xiao

**Affiliations:** Department of Social Medicine and Health Management, Xiangya School of Public Health, Central South University, Changsha 410078, China; angela_niulu@hotmail.com (L.N.); 146911042@csu.edu.cn (Y.L.); xiaosy@csu.edu.cn (S.X.)

**Keywords:** HIV/AIDS, websites, evaluation, quality

## Abstract

*Objective*: The present study was designed to assess the quality of Chinese-language Internet-based information on HIV/AIDS. *Methods*: We entered the following search terms, in Chinese, into Baidu and Sogou: “HIV/AIDS”, “symptoms”, and “treatment”, and evaluated the first 50 hits of each query using the Minervation validation instrument (LIDA tool) and DISCERN instrument. *Results*: Of the 900 hits identified, 85 websites were included in this study. The overall score of the LIDA tool was 63.7%; the mean score of accessibility, usability, and reliability was 82.2%, 71.5%, and 27.3%, respectively. Of the top 15 sites according to the LIDA score, the mean DISCERN score was calculated at 43.1 (95% confidence intervals (CI) = 37.7–49.5). Noncommercial websites showed higher DISCERN scores than commercial websites; whereas commercial websites were more likely to be found in the first 20 links obtained from each search engine than the noncommercial websites. *Conclusions*: In general, the HIV/AIDS related Chinese-language websites have poor reliability, although their accessibility and usability are fair. In addition, the treatment information presented on Chinese-language websites is far from sufficient. There is an imperative need for professionals and specialized institutes to improve the comprehensiveness of web-based information related to HIV/AIDS.

## 1. Introduction

Since 2011, “Getting to zero” has been the annual World AIDS Day theme, which reflects the ambitious aim of the entire world to end the AIDS epidemic. The world has progressed in halting and reversing the epidemic of HIV, which is still a primary cause of morbidity and mortality globally [1]. In China, although the national prevalence of HIV/AIDS remains low, the number of people living with HIV continues to increase [2,3]. At the end of October 2015, the number of reported cases of HIV-positive people in China increased to 575,000 [2] from 272,000 in 2009 [3]. It was estimated that there were 780,000 individuals infected by HIV at the end of 2011 in China [4]. This indicates that almost one-third are still unidentifed. Thus, there is still a long way to go to succeed in “getting to zero” in China.

To inform people about HIV/AIDS is imperative, no matter at universal, selected or indicated levels of preventions and interventions. For example, at the universal level, public education campaigns about the transmission, symptoms, treatment options, and prognosis for HIV/AIDS helps shift the social norms and decrease stigma of people living with HIV [5]. At the selected level, knowledge is a prerequisite for people with high HIV risk to change their risky behaviors [6] and intensify HIV testing [7]. At the indicated level, the knowledge is important for people living with HIV to make the right decisions about their health [8], such as adhering to proper care and maintaining good treatment adherence.

Nowadays, the Internet has become one of the major sources for people to meet their informational needs [9,10]. Up to 30 June 2015, there were 668 million Chinese Internet users, and over 594 million people were using their mobiles to rapidly access online information [11]. Health and medical treatment has been the most popular science theme in China [12]. Previous studies found that most of the key population included Internet users, and the majority were willing to attend online interventions [13,14]. A study, with a sample of 1022 female sex workers in Southwest China, showed that 75% of the sex workers were Internet users, among which 40% had searched HIV/STI (sexually transmitted infection) information online; and two thirds were willing to participate in an online HIV/STI prevention program [13]. Another study, with a sample of 1324 Chinese men who have sex with men, found that the majority used computer or mobile phones to search online information on HIV/STDs (sexually transmitted diseases), and most of them were interested in using eHealth interventions for sexual health [14]. It suggests that the Internet has great potential for health professionals to spread accurate HIV/AIDS-related knowledge and conduct effective prevention and intervention programs among key populations. Moreover, because of HIV-related stigma, discrimination, and possible shame related with seeking help, people with diagnosed or undiagnosed HIV infection and their relatives may search for good quality information on the Web to fulfill their needs [15].

Presently, there is an overwhelming amount of Web-based information related to HIV available to the public. Searching the web with the keyword “AIDS” (in Chinese) results in more than 44,800,000 hits (Baidu.com; July 2015). Such considerable amounts of information could easily overwhelm people living with HIV [16]. Further, although the Internet has the potential to educate people, inaccurate Web-based information may negatively affect people’s beliefs and behaviors, particularly of those with lower literacy levels and socioeconomic status [17,18,19]. For example, a study found that people living with HIV who used Internet more frequently were more likely to have treatment denialism beliefs, such as “HIV treatments do more harm than good” and “herbal and natural remedies can cure AIDS in some people” [20]. Such denialism beliefs were associated with refusing HIV treatments and poorer health outcomes [20]. Additionally, inaccurate information can also increase HIV-related discrimination and stigma.

Currently, as far as the authors of this study could ascertain, no study has evaluated the quality of any HIV-related websites. Thus, the present study aimed to: assess the accessibility, usability, and reliability of Chinese-language HIV/AIDS-related information available on the Internet; evaluate the quality of online information on HIV/AIDS treatment; and share our thoughts of important future directions for managing the information on Chinese-language websites regarding HIV/AIDS.

## 2. Methods

### 2.1. Selection of Websites

We conducted keyword searches from a China (mainland) based IP address in July 2015. The following sets of Chinese keywords were respectively entered into two of the most commonly used Chinese-language search engines, Baidu and Sogou based on Alexa Traffic Ranks (updated on 13 July 2015) [21]:

(1) “Acquired immunodeficiency syndrome”, “Acquired immunodeficiency syndrome symptom”, “Acquired immunodeficiency syndrome treatment”;
(2) “AIDS”, “AIDS symptom”, “AIDS treatment”;
(3) “HIV”, “HIV symptom”, “HIV treatment”.

We screened the first 50 hits from each search engine for each keyword request. Duplicated links to the same website were excluded, along with news groups, journal articles, blogs, open forum sites or open sharing platform, audio links, and video sites.

### 2.2. Evaluation of Websites

We divided the websites into five categories according to the suffix and their affiliation statement: commercial, governmental, academic, hospital and others (e.g., non-governmental organization or private).

The Health on the Net code (HONcode) is a seal of approval that is attributed to medical websites, which adheres to the following eight principles defined in the HONcode charter: authoritative, complementarity, privacy, attribution, justifiability, transparency, financial disclosure, and advertising policy [22]. Currently, nearly 7000 sites in 52 languages are certified. We used the HON toolbar, a plug-in of Mozilla Firefox (Mozilla, Mountain View, CA, USA), to identify the HONcode-certified websites [23].

We used the Minervation validation instrument (LIDA tool) for health care websites to assess accessibility, usability, and reliability of websites [24]. The LIDA tool is an online validation instrument [25], which consists of an automated test of accessibility and nine questions of usability and reliability. The LIDA accessibility assessment determines whether a website can be accessed by users and conforms to legal accessibility standards (i.e., standards proposed by the World Wide Web Consortium; W3C) [26]. The LIDA usability assesses whether the information is designed and presented in a manner to allow users to obtain what they want [26]. The third facet of the LIDA tool is to assess information reliability on a website in terms of currency (updated regularly), conflicts of interest (disclosure of sponsorship and objectives), and content production (quality control, source and references) [26]. Each question is rated on a 4-point Liker scale (0 = never; 3 = always) with higher scores indicating better quality of websites, and the overall score is calculated as a percentage. A score of >90% represents good quality, whereas a score lower than 50% indicates poor quality [27].

Next, we used the DISCERN instrument to evaluate the top 15 results from the LIDA tool. The DISCERN instrument includes 16 items on a 5-point scale (1 = not at all; 5 = completely) [28]. The first eight items evaluate the reliability of content, and the next seven estimate the quality of treatment information. The last item is an overall quality rating. The total score ranges from 16 to 80, and a higher score indicates better website-content quality.

The websites were evaluated by two reviewers (LN and YL) independently. Wherever the ratings differed between the two reviewers, the reviewers attempted to arrive at a consensus for a rating by mutual discussion. All websites in which such consensus could not be reached were referred to a senior reviewer (DL).

### 2.3. Statistical Analysis

Statistical analyses were performed by using SPSS for Windows (version 17.0; IBM Corporation, Armonk, NY, USA). We calculated means, standard deviations, 95% confidence intervals (CI), and score ranges of LIDA and DISCERN tools. Mann–Whitney tests were conducted to test whether the DISCERN scores of the affiliation groups in the top 15 websites (according to LIDA score) differed, and a significance level of *p* ≤ 0.05 was used.

## 3. Results

### 3.1. General Characteristics of Websites

We reviewed 900 hits, of which 754 were excluded for being duplicated websites. From the remaining 146 websites, 61 were excluded because 30 were news groups, 26 were open forum sites, two were open sharing platforms, one was a journal article database, and two were inaccessible links. Therefore, 85 websites were included. The sites were owned by primarily commercial organizations (63/85, 74.1%), and only one was certified with the HONcode (WHO website, in Chinese [29]).

### 3.2. LIDA

Table 1 shows that the average LIDA score for all websites was 63.7% (95% CI = 62.0%–65.3%, range 42.7%–82.3%): 81 of 85 websites (95.3%) were categorized as moderate, while the other four (4.7%) were categorized as poor.

The mean *Accessibility* rating was 82.2% (95% CI = 80.2%–84.0%, range 55.6%–100%). The majority (71/85, 83.5%) of websites were categorized as moderate, and the remainder (14/85, 16.5%) were rated as good.

The mean *Usability* score was 71.5% (95% CI = 68.9%–74.1%, range 50.0%–100%); 10.6% (9/85) of websites were categorized as good, 82.4% (76/85) as moderate, and 7.1% as poor.

The *Reliability* score were highly variable and ranged from 0% to 73.3%, with a mean of 27.3% (95% CI = 23.9%–31.1%): 10 of 85 (11.8%) websites were scored within the moderate range, while the majority (75/85, 88.2%) were categorized as poor. The majority of the websites either did not report a robust quality control procedure (80/85, 94.1%); were not checked by an expert (75/85, 88.2%); or did not cite relevant sources (76/85, 89.4%).

### 3.3. Characteristics of the Top 15 Websites

Among the top 15 websites according to LIDA scores, 10 were commercial, one was government authored, and the other four belonged to NGO or nonprofit organizations. In addition, 66.7% (10/15) websites were found on both Baidu and Sogou, while 33.3% (5/15) were found only on Sogou. Compared to noncommercial websites, commercial websites were more likely to be found in the first 20 links (in page 1 or 2) of the search results of both search engines (Table 2 and Table 3).

### 3.4. DISCERN

The DISCERN scores of the top 15 websites (Table 4) were also highly variable and ranged from 32 to 76, with a mean of 43.1 (95% CI = 37.7–49.5). The mean scores of subscales were as follows: reliability 25.1 (95% CI = 22.5–27.8, range 18–36), treatment information 15.0 (95% CI = 11.4–19.5, range 7–35), and the overall quality rating 3.0 (95% CI = 2.5–3.5, range 2–5).

In terms of reliability, over half of websites had poor reliability (≤2) regarding explicit source (8/15, 53.3%), explicit date (9/15, 60.0%), balance and unbiasedness (8/15, 53.3%), areas of uncertainty (10/15, 66.7%).

Moreover, the majority of websites had poor reliability (≤2) in terms of treatment information regarding how treatment works (9/15, 60.0%), treatment risks (11/15, 73.3%), effects of no treatment (12/15, 80.0%), effects on quality of life (13/15, 86.7%), and shared decision (11/15, 73.3%).

Commercial websites were likely to have lower overall DISCERN score (37.1 ± 3.5 vs. 55.2 ± 15.5, *p* = 0.003) and lower reliability (21.9 ± 2.9 vs. 31.6 ± 3.2, *p* = 0.003) than noncommercial websites. However, the score of treatment information did not significantly differ between commercial and noncommercial websites (12.6 ± 3.10 vs. 19.8 ± 13.16, *p* = 0.580).

## 4. Discussion

To our knowledge, this is the first study to investigate the current status of Chinese-language websites containing information about HIV/AIDS. In our review of 85 websites, only one was certified with the HONcode (WHO website [29]). Similarly, Zhang et al. evaluated the 20 most popular health information-related websites in China in 2012, and none of them was HONcode certified [44]. This may because of two reasons. The first reason is that the HONcode certification may not have been well known in China then; thus, many Chinese health related websites did not apply for the HONcode certification. The second most important reason is that most of the included websites may not be qualified for a HONcode certification, as the results of the LIDA tool and DISCERN instrument indicate in the current study regarding some specific quality issues in the online HIV/AIDS information.

According to the LIDA criteria, major websites are easily accessible and usable. However, of particular concern is reliability, especially when most websites were not checked by an expert, and did not mention quality control procedures and references of the content. It raises questions about the trustworthiness of the websites because reliable information should be generally prepared or checked by experts of a particular field, and any conflicts of interest should be minimized or disclosed on the website [26,45].

Furthermore, the DISCERN tool was used to evaluate the quality of treatment information of the top 15 websites according to their LIDA scores. The results showed poor reliability of these websites because most of them did not have explicit dates of publication and sources of information and did not refer to areas of uncertainty. Regarding the information on treatment, over half of the top 15 sites had poor reliability according to the DISCERN criteria.

Our results suggest that people may face difficulties in finding trustworthy and complete information on HIV/AIDS using the Internet. According to the information-motivation-behavior skills model, information is one of the fundamental determinants of preventive behavior [6], and a prerequisite of health behavior change [46]. In terms of HIV prevention, users are well informed about HIV transmission and prevention, and are motivated to engage in HIV-risk-reduction behavior (e.g., use of condoms). Thus, they are more likely to develop and use the related behavioral skills and engage in preventive behavior [6]. Therefore, the unreliable or biased information online may lead to less favorable attitudes about HIV preventive behavior, and negatively influence the utilization of behavioral skills and enactment of risk-reduction behavior. Besides, if people rely on the Internet to make treatment decisions, including whether to get an HIV test, seek help, and retain in health care system, inaccurate information and deficiencies in information could negatively influence people’s decisions and behavior [47]. For example, it is known that the efficiency of antiretroviral therapy (ART) depends upon people living with HIV having a sustained 95% adherence rate to their regimen for their lifetime [48]. If they cannot maintain this adherence level, their body will become resistant to treatment [48] and lead to poor HIV outcomes [49]. Thus, information including life quality issues, treatment risks, and effects of no treatment will help HIV-positive people be prepared for treatment and maintain good adherence after ART [50]. However, these three aspects had the lowest scoring according to the DISCERN tool in this study.

Despite the generally poor scores for reliability and treatment-related information of the included websites, we did recognize some sites with reliable and important information about HIV/AIDS, such as the WHO website [29], Chinese Chain Net (China HIV/AIDS Information Network) [30], and website of Chinese Center of Disease Control and Prevention (CDC) [33]. However, these websites could rarely be found in the first 20 links of the search results, especially in the Baidu search engine, because most of them are noncommercial. Given that most people rarely look beyond the first 20 links returned from a search [51], it is less likely for these websites to be accessed.

The present study has a few limitations. First, the results of this study only reflect the situation of HIV/AIDS-related websites active in July 2015. Second, the search methods (i.e., the search engines and search terms) we used to identify websites do not include all the information resources that people may utilize. Third, there may be useful information in the excluded websites, such as news groups, open forum sites or open sharing platform, and video sites. Future studies are needed to evaluate these information resources. Fourth, integration of health-care providing experts (e.g., physicians and professionals in CDCs), people living with HIV, and the general population into the evaluation team may add new perspectives and improve quality of evaluation in future studies.

## 5. Conclusions

In conclusion, HIV/AIDS-related Chinese-language websites have low reliability in general, although their accessibility and usability are fair. In addition, the treatment information presented on such websites is not adequate. To improve Chinese web-based HIV/AIDS information is an urgent necessity, and requires collaboration between health professionals and website developers and managers. First, it needs to be ensured that the information is reliable in implementing quality control, citing reference or sources for information, and including a publication date. Second, the focus should be on providing more complete information on prevention, testing, and diagnosis of HIV as well as treatment-related information. Third, future studies should shed light on leading HIV-positive people and the public to better quality websites currently available, and on developing an integrated working mechanism among the stakeholders, such as health professionals, website developers, and health administrative departments, and help patients and the public make better use of Internet resources.

## Figures and Tables

**Table 1:** Scores of the included websites (*N* = 85) measured by the LIDA tool.

Section	Questions	Mean (SD)	Range
Accessibilty	(automated test)	82.2% (8.9%)	55.6%–100%
Usability		71.5% (11.8%)	50.0%–100%
	Is the site design clear and transparent?	2.2 (0.7)	1–3
	Is the site design consistent from one page to another?	3.0 (0.1)	2–3
	Can users find what they need on the site?	1.5 (0.5)	1–3
	Is the format of information clear and appropriate for the audience?	1.9 (0.5)	1–3
Reliability		27.3% (16.7%)	0%–73.3%
	Is it clear who has developed the web site and what their objectives are?	2.2 (1.1)	0–3
	Does the site report a robust quality control procedure?	0.2 (0.7)	0–3
	Is the page content checked by an expert?	0.3 (0.8)	0–3
	Is the page updated regularly?	1.3 (1.0)	0–3
	Does the page cite relevant sources where appropriate?	0.2 (0.6)	0–3
Total score		63.7% (8.0%)	42.7%–82.3%

**Table 2:** The searching results of the top 15 websites obtain from Baidu by each query.

Websites ^&^	Affilation ^#^	Search Terms (Page *)									Frequency in the First Two Pages
		Acquired Immunodeficiency Syndrome	Acquired Immunodeficiency Syndrome Symptom	Acquired Immunodeficiency Syndrome Treatment	AIDS	AIDS Symptom	AIDS Treatment	HIV	HIV Symptom	HIV Treatment	
1. WHO [29]	N							4			0
2. Chain net [30]	N	5									0
3. Wikipedia [31]	N				1; 4 ^a^			4			1
4. Sogou Baike [32]	C	4		5	5 (2) ^b^						0
5. Chinese Center for Disease Control and Prevention [33]	N	4									0
6. Dermatologist [34]	N										0
7. Health First Line [35]	C										0
8. Baike [36]	C										0
9. FX120.net [37]	C	3	3	3		3	2	3		3	1
10. Baidu Baike [38]	C	1 (2); 2	1; 2	1	1 (2)	1; 2	1	1 (2); 2; 3	1 (2)	1	13
11. 99.com [39]	C	2		3	2		2	1; 2			5
12. Qiuyi [40]	C	4; 5	5	4; 5		4	3		4	1	1
13. Med66 [41]	C										0
14. XYWY.com [42]	C	1; 2; 3; 5 (2)	4; 5	1; 2; 4; 5	1; 5	4 (4)	4 (2); 5		1; 4; 5	1	7
15. JKPJ.com [43]	C										0

Note: * Each page showed 10 results. ^&^ The ranks of the websites were based on the DISCEN scores. ^#^ N = non-commercial; C = commercial. ^a^ 1; 4 means there was one link of the website in page 1 and page 4 respectively. ^b^ 5 (2) means there were two links of the website in page five.

**Table 3:** The searching results of the top 15 websites obtain from Sogou by each query.

Websites ^&^	Affilation ^#^	Searching terms (Page *)									Frequency in the First Two Pages
		Acquired Immunodeficiency Syndrome	Acquired Immunodeficiency Syndrome Symptom	Acquired Immunodeficiency Syndrome Treatment	AIDS	AIDS Symptom	AIDS Treatment	HIV	HIV Symptom	HIV Treatment	
1. WHO [29]	N				1; 2 ^a^			1; 2			4
2. Chain net [30]	N				5						0
3. Wikipedia [31]	N				1; 2 (2) ^b^			1; 2			5
4. Sogou Baike [32]	C	1		1; 3; 4	1		2; 4	1		1	6
5. Chinese Center for Disease Control and Prevention [33]	N				3						0
6. Dermatologist [34]	N									5	0
7. Health First Line [35]	C	1	1	1				1	1	1	6
8. Baike [36]	C	2 (2)								4	2
9. FX120.NET [37]	C	2	2			4	4				2
10. Baidu Baike [38]	C			4	3				1	4	1
11. 99.com [39]	C	5		2; 3		5	1; 3				2
12. Qiuyi [40]	C	1 (2); 5	1 (2)	1 (3); 2 (3)	1 (2)	1	1 (2); 5 (2)			1	16
13. Med66 [41]	C			1		4					1
14. XYWY.com [42]	C	1; 5 (2)	1; 2; 5	1 (2); 3	2	1; 2; 4	1; 5		2; 5 (2)		11
15. JKPJ.com [43]	C									3	0

Note. * Each page showed 10 results. ^&^ The ranks of the websites were based on the DISCEN scores. ^#^ N = non-commercial; C = commercial. ^a^ 1; 2 means there was one link of the website in page 1 and page 2 respectively. ^b^ 2 (2) means there were two links of the website in page 2.

**Table 4:** Scores of the top 15 websites measured by the DISCERN instrument.

Sections	Items	Mean (SD)	Range
Reliability		25.1 (5.5)	18–36
	Explicit aims	5.0 (0)	5–5
	Aim achieved	3.7 (0.7)	3–5
	Relevance	3.6 (0.7)	2–5
	Explicit sources	2.4 (1.4)	1–5
	Explicit date	2.6 (1.1)	1–5
	Balanced and unbiased	2.7 (1.3)	1–5
	Additional sources	2.8 (0.9)	1–4
	Ares of uncertainty	2.3 (1.2)	1–5
Treatment options		15.0 (8.2)	7–35
	How treatment works	2.3 (1.2)	1–5
	Benefits of treatment	2.7 (1.1)	1–5
	Risks of treatment	1.9 (1.4)	1–5
	Effects of no treatment	1.9 (1.4)	1–5
	Effects on quality of life	1.5 (1.4)	1–5
	All options described	2.7 (1.1)	1–5
	Shared decision	1.9 (1.5)	1–5
Overall rating		3.0 (1.0)	2–5
Total score		43.1 (12.4)	32–76

## References

[1] Suthar A.B., Ford N., Bachanas P.J., Wong V.J., Rajan J.S., Saltzman A.K., Ajose O., Fakoya A.O., Granich R.M., Negussie E.K. (2013). Towards universal voluntary HIV testing and counselling: A systematic review and meta-analysis of community-based approaches. PLoS Med..

[2] Xinhua Net There Were Approximately 575,000 People Living with HIV in China and the Proportion of Male Homosexual Transmition Siginifcantly Increased. http://news.xinhuanet.com/health/2015-11/30/c_1117308884.htm.

[3] National Health and Family Planning Commission of China 2014 China AIDS Response Progress Report. http://www.unaids.org.cn/cn/index/Document_view.asp?id=860.

[4] The Former Chinese Ministry of Health, UNAIDS and WHO 2011 China AIDS Response Progress Report. http://ncaids.chinacdc.cn/fzdt/zxdd/201201/t20120129_1745902.htm.

[5] Stangl A.L., Lloyd J.K., Brady L.M., Holland C.E., Baral S. (2013). A systematic review of interventions to reduce HIV-related stigma and discrimination from 2002 to 2013: How far have we come?. J. Int. AIDS Soc..

[6] Fisher J.D., Fisher W.A. (1992). Changing aids-risk behavior. Psychol. Bull..

[7] Hanf M., Bousser V., Parriault M.C., Van-Melle A., Nouvellet M.-L., Adriouch L., Sebillotte C.G., Couppie P., Nacher M. (2011). Knowledge of free voluntary HIV testing centres and willingness to do a test among migrants in Cayenne, French Guiana. AIDS Care.

[8] Edewor N. (2010). Access to health information by people living with HIV/AIDS in Nigeria. Libr. Philos. Pract..

[9] Kremer H., Ironson G. (2007). People living with HIV: Sources of information on antiretroviral treatment and preferences for involvement in treatment decision-making. Eur. J. Med. Res..

[10] Curioso W.H., Kurth A.E. (2007). Access, use and perceptions regarding internet, cell phones and PDAs as a means for health promotion for people living with HIV in Peru. BMC Med. Inform. Decis. Mak..

[11] China Internet Network Information Center Basic Data. http://www.cnnic.net.cn/hlwfzyj/jcsj2014/201507/t20150723_52639.htm.

[12] China Science Communication Report on Chinese Netizens’ Need and Search Behaviors of Science Communication, the Second Season. http://index.baidu.com/special/kepu/.

[13] Hong Y., Li X., Fang X., Lin X., Zhang C. (2011). Internet use among female sex workers in China: Implication for HIV/STI prevention. AIDS Behav..

[14] Muessig K.E., Bien C.H., Wei C., Lo E.J., Yang M., Tucker J.D., Yang L., Meng G., Hightow-Weidman L.B. (2015). A mixed-methods study on the acceptability of using eHealth for HIV prevention and sexual health care among men who have sex with men in China. J. Med. Internet Res..

[15] Courtenay-Quirk C., Horvath K.J., Ding H., Fisher H., Mcfarlane M., Kachur R., O’Leary A., Rosser S.B.R., Harwood E. (2010). Perceptions of HIV-related websites among persons recently diagnosed with HIV. AIDS Patient Care STDS.

[16] Hogan T.P., Palmer C.L. (2005). Information preferences and practices among people living with HIV/AIDS. Results from a nationwide survey. J. Med. Libr. Assoc..

[17] Benotsch E.G., Kalichman S., Weinhardt L.S. (2004). HIV-AIDS patients’ evaluation of health information on the internet: The digital divide and vulnerability to fraudulent claims. J. Consult. Clin. Psychol..

[18] Kalichman S.C., Cherry C., Cain D., Weinhardt L.S., Benotsch E., Pope H., Kailchman M. (2006). Health information on the Internet and people living with HIV/AIDS: Information Evaluation and Coping styles. Health Psychol..

[19] Chen S., Yang N., Zhu J. (2009). A study on the Internet use among MSM in Nanning. Chin. J. Health Educ..

[20] Kalichman S.C., Eaton L., Cherry C. (2010). “There is no proof that HIV causes AIDS”: AIDS denialism beliefs among people living with HIV/AIDS. J. Behav. Med..

[21] China Webmaster. http://top.chinaz.com/top500?t=254&fn=alexa.

[22] HONcode Health on the Net Foundation. http://www.hon.ch/HONcode/Conduct.html.

[23] HONtools Health on the Net Foundation. http://www.hon.ch/HONtools/Patients/Plugin/Plugins.html.

[24] MINERVATION. http://www.minervation.com/lida-tool/.

[25] LIDA Tool (Online Validation) MINERVATION. http://lida.minervation.com/Validation.aspx.

[26] Full LIDA Tool (v1.2 Copyright Minervation 2007). http://www.minervation.com/lida-tool/minervation-lida-instrument-v1-2/.

[27] Hendrick P.A., Ahmed O.H., Bankier S.S., Chan T.J., Crawford S.A., Ryder C.R., Welsh L.J., Schneiders A.G. (2012). Acute low back pain information online: An evaluation of quality, content accuracy and readability of related websites. Man. Ther..

[28] DISCERN: General Instructions. http://www.discern.org.uk/general_instructions.php.

[29] World Health Organization HIV/AIDS. http://www.who.int/hiv/zh/.

[30] China HIV/AIDS Information Network. http://new.chain.net.cn/index.html.

[31] HIV/AIDS from Wikipedia. https://zh.wikipedia.org/wiki/%E8%89%BE%E6%BB%8B%E7%97%85.

[32] Sogou Baike: AIDS Prevention Knowledge. http://baike.sogou.com/v54230195.htm.

[33] Chinese Center for Disease Control and Prevention. http://www.chinacdc.cn/.

[34] Dermatologist. http://www.anaids.com/china-first-line-treatment-of-hiv.html.

[35] Health First Line: AIDS. http://crk.vodjk.com/azb/.

[36] Interation Baike: HIV. http://www.baike.com/wiki/HIV.

[37] AIDS. http://www.fx120.net/disease5/azb/.

[38] Baidu Baike: AIDS. http://baike.baidu.com/link?url=b6ihhaAaQmCzhizU7r2F4N1yKXsi1D4M4FxZkabWyfvHUeA2B_Z02wXvuICiNY2O.

[39] AIDS. http://www.99.com.cn/azb/.

[40] AIDS. http://jibing.qiuyi.cn/azb/.

[41] Symptoms of AIDS. http://www.med66.com/html/2008/9/wa51250191028980022530.html.

[42] AIDS. http://aids.xywy.com/.

[43] HIV Treatment New Guidelines. http://www.jkpj.com/jibin/xinbin/hiv/200705/4342.html.

[44] Zhang H., Ma J., Jiang C., Zhou Y., Qiu J. (2013). Assessment research on information quality of health websites. J. Med. Inform..

[45] Feghhi D.P., Komlos D., Agarwal N., Sabharwal S. (2014). Quality of online pediatric orthopaedic education materials. J. Bone Jt. Surg. Am. Vol..

[46] Cai Y., Ye X., Shi R., Shen L., Ren J., Huang H. (2013). Predictors of consistent condom use based on the Information-Motivation-Behavior Skill (IMB) model among senior high school students in three coastal citeis in China. BMC Infect. Dis..

[47] Berland G.K., Elliott M.N., Morales L.S., Algazy J.I., Kravitz R.L., Broder M.S., Kanouse D.E., Munoz J.A., Puyol J.A., Lara M. (2001). Health information on the internet: Accessibility, quality, and readability in English and Spanish. JAMA.

[48] Schaecher K.L. (2013). The importance of treatment adherence in HIV. Am. J. Manag. Care.

[49] Jones D., Cook R., Rodriguez A., Waldrop-Valverde D. (2013). Personal HIV knowledge, appointment adherence and HIV outcomes. AIDS Behav..

[50] Kalichman S., Pellowski J., Chen Y. (2013). Requesting help to understand medical information among people living with HIV and poor health literacy. AIDS Patient Care STDs.

[51] Klila H., Chatton A., Zermatten A., Khan R., Preisig M., Khazaal Y. (2013). Quality of web-based information on obsessive compulsive disorder. Neuropsychiatr. Dis. Ttreat..

